# Alterations in Fecal Microbiota Linked to Environment and Sex in Red Deer (*Cervus elaphus*)

**DOI:** 10.3390/ani13050929

**Published:** 2023-03-04

**Authors:** Yue Sun, Yanze Yu, Jinhao Guo, Linqiang Zhong, Minghai Zhang

**Affiliations:** 1 School of Biological Sciences, Guizhou Education University, Guiyang 550018, China; 2 Wildlife Institute of Heilongjiang Province, Harbin 150081, China; 3 College of Wildlife and Protected Area, Northeast Forestry University, Harbin 150040, China; 4 College of Life Sciences and Technology, Xinjiang University, Urumqi 830046, China

**Keywords:** cervidae, KEGG, fecal microbiota, 16s rRNA

## Abstract

**Simple Summary:**

The gut microbiota forms a complex microecosystem in vertebrates and is affected by various factors. Wild and captive red deer currently live in the same region but have vastly different diets. In this study, the 16S rRNA sequencing technology was performed to evaluate variations in the fecal microbiota of wild and captive individuals of both sexes of red deer. It was found that the composition and function of fecal microbiota in wild and captive environments were significantly different. As a key intrinsic factor, sex has a persistent impact on the formation and development of gut microbiota. Overall, this study reveals differences in the in the fecal microbiota of red deer based on environment and sex. These data could guide future applications of population management in red deer conservation.

**Abstract:**

Gut microbiota play an important role in impacting the host’s metabolism, immunity, speciation, and many other functions. How sex and environment affect the structure and function of fecal microbiota in red deer (*Cervus elaphus*) is still unclear, particularly with regard to the intake of different diets. In this study, non-invasive molecular sexing techniques were used to determine the sex of fecal samples from both wild and captive red deer during the overwintering period. Fecal microbiota composition and diversity analyses were performed using amplicons from the V4–V5 region of the 16S rRNA gene sequenced on the Illumina HiSeq platform. Based on Picrust2 prediction software, potential function distribution information was evaluated by comparing the Kyoto Encyclopedia of Genes and Genome (KEGG). The results showed that the fecal microbiota of the wild deer (WF, *n* = 10; WM, *n* = 12) was significantly enriched in Firmicutes and decreased in Bacteroidetes, while the captive deer (CF, *n* = 8; CM, *n* = 3) had a significantly higher number of Bacteroidetes. The dominant species of fecal microbiota in the wild and captive red deer were similar at the genus level. The alpha diversity index shows significant difference in fecal microbiota diversity between the males and females in wild deer (*p* < 0.05). Beta diversity shows significant inter-group differences between wild and captive deer (*p* < 0.05) but no significant differences between female and male in wild or captive deer. The metabolism was the most important pathway at the first level of KEGG pathway analysis. In the secondary pathway of metabolism, glycan biosynthesis and metabolism, energy metabolism, and the metabolism of other amino acids were significantly different. In summary, these compositional and functional variations in the fecal microbiota of red deer may be helpful for guiding conservation management and policy decision-making, providing important information for future applications of population management and conservation.

## 1. Introduction

Red deer (*Cervus elaphus*), which belong to the family Cervidae, order Artiodactyla, distributed in Asia, Europe, North America, and North Africa [1]. The red deer is a typical forest-inhabiting mammal in northeast China and has an important ecological status in the forest ecosystem [2]. Owing to habitat fragmentation, the populations of red deer in the wild are currently in sharp decline [2]. Using captive populations as reintroduction resources is an effective strategy to restore the populations of wild red deer [3].

The complex gut microbiota systems in the mammalian gut are composed of large fractions of microbes [4]. The gut microbiota are a complex product of the long-term evolution of hosts and microbes [4]. Recent studies have shown that not only are gut microbiota a part of the host, but they also have a significant impact on the health of the host, such as promoting immunity, digestion, metabolism, and intestinal endocrine hormones, among others [5,6,7]. Simultaneously, the complex and flexible gut microbiota can be affected by multiple environmental and host genotypes [8]. Many studies have shown that diet is an important factor that affects the structure and function of the fecal microbiota [9,10,11]. For example, changes in diet alter the function and diversity of fecal microbiota as well as the relative abundance of some microorganisms [12]. Moreover, diet-induced loss of microbial function and diversity will increase the risk of diversity loss and extinction through generational amplification [13]. It was necessary to investigate the gut microbiome by comparing differences between wild and captive red deer. However, to date, there has been a lack of studies comparing the gut microbiota between wild and captive red deer [11]. Because of sex differences in behavior and physiology, sex as an important intrinsic factor leads to differences in gut microbiota among individuals within species [14,15,16]. Although the results are inconsistent, animal species with significant sexual dimorphism and human studies have shown sex-related differences in gut microbiota. In mice (*Mus musculus*), poultry, and forest musk deer (*Moschus berezovskii*), the composition of the gut or fecal microbiota shows sex differences [17,18,19]. At present, few studies have analyzed the sexual dimorphism of fecal microbiota in red deer.

In order to save endangered populations, artificial breeding of wild populations is carried out. The food types and nutrient intake ratios obtained in captivity and wild environments are very different, especially for endangered cervidae [20]. Therefore, monitoring the digestive system of captive animals and identifying standardized levels of nutritional requirements and fiber composition is critical for captive wild animals to determine whether they have acclimated to artificially provided food and new environments—a part of wildlife conservation’s main problem [21]. Using captive populations as reintroduction resources is an effective strategy to restore the populations of wild red deer. The composition of gut microbiota in wild populations can be a good indicator of the breeding direction of the captive population [9]. Therefore, understanding the impact of dietary differences between wild and captive red deer on the fecal microbiota can help to assess and ensure the long-term viability of this species [9]. At present, the research methods for fecal microbiota have also shifted from traditional methods to 16S rRNA gene sequencing technology, from simple microbial composition, community structure, and core microbiota research to microbial function research, which has become a hot frontier in ungulate research today [22]. 

The main goal of this study was to characterize the composition of the fecal microbiota of red deer of different sex and feeding plus environment. We used high-throughput 16S rRNA sequencing technology to comprehensively analyze. Thus, we hypothesized that: (1) the fecal microbiota composition and function are different between wild and captive deer; and (2) under the wild or captive environment, the microbiota diversity and evenness are different between females and males.

## 2. Materials and Methods

### 2.1. Study Site, Subjects, and Sample Collection

This study was conducted at the Gaogestai National Nature Reserve in Chifeng, Inner Mongolia (119°02′30″, 119°39′08″ E; 44°41′03″, 45°08′44″ N). The total area is 106,284 hm^2^. It is a typical transition zone forest-steppe ecosystem in the southern foothills of Greater Khingan Mountains, including forests, shrubs, grasslands, wetlands, and other diverse ecosystems. In February 2019, 75 line transects were randomly laid in the Gogestai protection area. Positive and reverse footprint chain tracking was carried out after the foodprints of red deer were found through line transect investigation. Disposable PE gloves were worn to collect red deer feces. While tracking the footprint chain, set 2 m × 2 m plant quadrate every 200 m to 250 m along the footprint chain, and collect all kinds of plant branches eaten by deer in the quadrate as far as possible [23]. A total of 162 fecal samples were collected and stored at −20 °C within 2 h. The feces of red deer from different areas of the Reserve were identified as coming from different individuals, and 43 feces were identified individually in the laboratory. 

In February 2019, the HanShan Forest Farm in Chifeng City, Inner Mongolia, China (adjacent to the Gaogestai Nature Reserve) had a total of 11 healthy adult red deer of similar age and size. Ear tags were used to differentiate each individual red deer. Through continuous observation, feces were collected immediately after excretion by different red deer individuals and stored at −20 °C. We measured crude protein, energy, neutral detergent fiber (NDF), and total non-structural carbohydrates in red deer diets. 

### 2.2. Individual Recognition and Sex Identification

We used a qiaamp DNA Fecal Mini-kit (QIAGEN, Hilden, Germany) to extract host deoxyribonucleic acid (DNA) from the fecal samples of red deer as previously described [24]. Microsatellite PCR technology was used with nine pairs of microsatellite primers (BM848, BMC1009, BM757, T108, T507, T530, DarAE129, BM1706, and ILST0S058) [25,26] with good polymorphism that were selected based on the research results of previous studies. These nine pairs of primers can amplify fecal DNA stably and efficiently. A fluorescence marker (TAMRA, HEX, or FAM) was added to the 5′ end of upstream primers at each site (Supplementary Table S1). Primer information, PCR amplification, and genotype identification procedures are described in the literature [27]. Multi-tube PCR amplification was used for genotyping [28], and 3–4 positive amplifications were performed for each locus to determine the final genotype [29]. The excel microsatellite toolkit [30] was used to search for matching genotypes from the data. Samples are judged to be from the same individual if all loci have the same genotype or if only one allele differs at a locus. The microsatellite data were analyzed by Cervus 3.0 software, and the genotyping was completed [31]. 

Male and female individuals were identified by detecting the existence of genes after the individual identification of red deer was completed. Sry gene primers (F:5′-3′ TGAACGCTTTCATTGTGTGGTC; R:5′-3′ GCCAGTAGTCTCTGTGCCTCCT) were designed, and the amplification system was determined. To minimize the occurrence of false positives or false negatives that could affect results, the Sry gene was repeated three times to expand and increase during the experiment, and samples with target bands that appeared on the second and third occasions were determined to be male [32].

### 2.3. Fecal Microbiota DNA Extraction, Amplification, and Sequencing

The total microbial DNA of fecal samples was extracted using an E.Z.N.A^®^ Soil DNA Kit (Omega Bio-Tek, Norcross, GA, USA). The DNA integrity of the extracted samples was determined by 1% agarose gel electrophoresis. Targeting a 420 bp fragment encompassing the V4-V5 region of the bacterial 16S ribosomal RNA gene was amplified by PCR using primers 515F (5′-GTG CCA GCM GCC GCG GTA A-3′) and 907R (5′-CCG TCA ATT CMT TTR AGT TT-3′). NEB 154 Q5 DNA high-fidelity polymerase (NEB, Ipswich, MA, USA) was used in PCR amplifications (Supplementary Table S1). A 1:1 mixture containing the same volume of 1XTAE buffer and the PCR products were loaded on a 2% agarose gel for electrophoretic detection. PCR products were mixed in equidensity ratios. Then, the mixture of PCR products was purified using the Quant-iTPicoGreen dsDNA Assay Kit (Invitrogen, Carlsbad, CA, USA). Sequencing libraries were generated using the TruSeq Nano DNA LT Library Prep kit (Illumina, San Diego, CA, USA) following the manufacturer’s recommendations, and index codes were added. The library’s quality was assessed on the Agilent 5400 (Agilent Technologies Co. Ltd., Santa Clara, CA, USA). At last, the library was sequenced on an Illumina NovaSeq 6000 platform, and 250 bp paired-end reads were generated.

Microbiome bioinformatics were performed with QIIME2 2019.4 [33] with slight modification according to the official tutorials (https://docs.qiime2.org/2019.4/tutorials/ (accessed on 30 September 2022)). Briefly, raw data FASTQ files were imported into the format that could be operated by the QIIME2 system using the qiime tools import program. The DADA2 [34] process is to obtain amplified variant sequences through de-duplication. In the process, clustering is not carried out based on similarity, but only de-duplication is carried out. Demultiplexed sequences from each sample were quality filtered and trimmed, de-noised, merged, and then the chimeric sequences were identified and removed using the QIIME2 DADA2 plugin to obtain the feature table of amplicon sequence variants (ASV) [34]. The QIIME2 feature-classifier plugin was then used to align ASV sequences to a pre-trained GREENGENES 13_8 99% database (trimmed to the V4V5 around a 420bp region bound by the 515F/907R primer pair) to generate the taxonomy table [35]. In order to unify the sequence effort, samples were rarefied at a depth of 25,318 sequences per sample before alpha and beta diversity analysis. Rarefaction allows one to randomly select a similar number of sequences from each sample to reach a unified depth. 

### 2.4. Bioinformatics and Statistical Analyses

Sequence data analyses were mainly performed using QIIME2 and R software (v3.2.0). ASV-level alpha diversity indices, such as the Chao1 richness estimator and Pielou’s evenness, were calculated using the ASV table in QIIME2 [36,37], and visualized as box plots (R software, package “ggplot2”). Beta diversity analysis was performed to investigate the structural variation of microbial communities across samples using weighted or unweighted UniFrac distance metrics [38,39] and visualized via principal coordinate analysis (PCoA) (R software, package “ape”). The significance of differentiation of microbiota structure among groups was assessed by PERMANOVA (permutational multivariate analysis of variance) [40]. Random forest analysis (R software, package “randomForest”) was applied to sort the importance of microbiota with differences in abundance between groups and screen the most critical phyla and genera that lead to microbial structural differences between groups using QIIME2 with default settings [41,42]. Phylogenetic Investigation of Communities by Reconstruction of Unobserved States (Picrust2) [43] is software that predicts the functional abundance from the sequencing data of marker genes (typically 16S rRNA). An ASV’s abundance table is used for standardization, and the corresponding relationship of each ASV is compared with the Kyoto Encyclopedia of Genes and Genomes (KEGG) library to obtain the functional information and functional abundance spectrum.

## 3. Results

### 3.1. Identification of Individuals and Sex

A total of 22 red deer individuals were identified from 43 fecal samples, including 12 males and 10 females (Supplementary Table S2). The female captive deer were CF1, CF2, CF3, CF4, CF5, CF6, CF7, and CF8. The male captive deer were CM1, CM2, and CM3. We divided all the red deer (22 wild and 11 captive) into four groups: wild females (WF) (*n* = 10), wild males (WM) (*n* = 12), captive females (CF) (*n* = 8), and captive males (CM) (*n* = 3). The information about identification, location, sex, and diet is summarized in Supplementary Table S2.

### 3.2. Diet Composition and Nutritional Composition of Wild and Captive Red Deer Winter Diets

The wild red deer were fed on 16 species of plants in the winter. The edible plants belonged to 16 species of 16 genera and 9 families. Since the frequency of occurrence of other edible plants in red deer, such as Mongolian oak (*Quercus mongolica*) and Chinese maple (*Acer sinensis*), was less than 7%, the nutrient content of these plants was not measured. In addition, we hypothesized that they had little influence on the nutritional strategy of red deer. Therefore, the primary nutrient contents of 14 types of edible plants were determined. The food and nutritional composition of wild red deer are shown in Supplementary Table S3. When the captive red deer were fed, each type of food was fed separately at different times. The nutritional content of the primary food of captive red deer from the farm (adjacent to the Gaogestai Nature Reserve) in winter is shown in Supplementary Table S4. Only one kind of diet were provided to captive deer at each feeding time with all captive deer feeding together. Captive red deer feed on leaves and high protein given by artificial feeding. Compared with captive red deer, wild deer have a wider feeding range and no dietary limitations. Substantial differences exist between these two feeding methods.

### 3.3. Sequencing Analysis and Clustering

A total of 1,561,654 high-quality sequences were obtained from the fresh winter feces of 22 wild deer and 11 captive deer. Rarefaction curves based on the Chao1 diversity index reached asymptotes at 22,500. The results showed that with the increase in amount of sequencing, the curve tended to be flat and no longer changed, indicating that the amount of sequencing in this study basically reflected the diversity of red deer fecal microbiota in this study (Supplementary Figure S1). A total of 15,228 ASVs were obtained using a 100% similarity clustering method. The WF, WM, CF, and CM groups included 3056 ASVs, 3924 ASVs, 6661 ASVs, and 1587 ASVs, respectively.

### 3.4. Microbial Composition and Diversity by Environment and Sex

**Figure 1:**
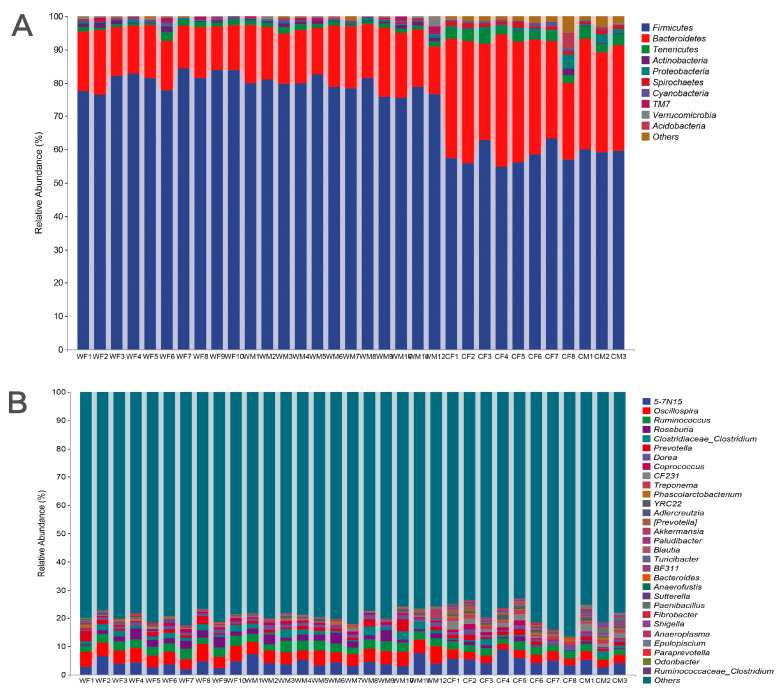
Relative abundance of the top 10 bacterial phyla and top 30 genera present in the fecal microbiota of wild and captive red deer (**A**) at the phylum level and (**B**) at the genus level (WF: *n* = 10, WM: *n* = 12, CF: *n* = 8, CM: *n* = 3).

We found significant differences in fecal microbial composition between wild and captive red deer based on environment. The fecal microbial communities of four groups (WF, WM, CF, and CM) were dominated by the phyla Firmicutes and Bacteroidetes (Figure 1A). The phylum Firmicutes was the most abundant in WF (81.12 ± 2.87%), followed by WM (79.03 ± 2.19%), CF (58.24 ± 3.17%), and CM (59.66 ± 0.47%). Secondly, Bacteroidetes was abundant in WF (15.19 ± 2.09), WM (16.89 ± 2.08%), CF (33.02 ± 5.48), and CM (31.55 ± 1.61%). At the genus level, the genera from the four groups with abundance > 1% were *Oscillospira*, a candidate genus *5-7N15* from the family Bacteroidaceae, *Ruminococcus*, *Roseburia*, *Clostridium*, and *Prevotella* (Figure 1B and Table 1).

**Figure 2:**
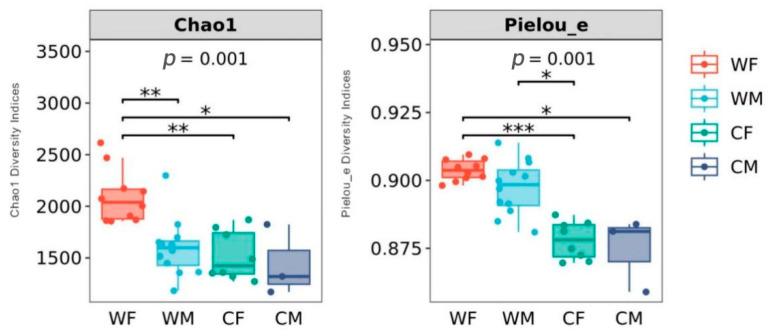
Boxplots of alpha-diversity indices showing differences between sex and environment (WF: wild female; WM: wild male; CF: captivity female; CM: captivity male). * represents significant (*p* < 0.05), ** represents very significant (*p* < 0.01), *** and represents highly significant (*p* < 0.001) (WF: *n* = 10, WM: *n* = 12, CF: *n* = 8, CM: *n* = 3).

The chao1 diversity indices demonstrate a significant difference between the WF and WM groups (*p* < 0.01). There was no statistically significant difference between the CF and CM groups (*p* > 0.05). Pieluo’s diversity index showed that no significant differences occurred between WF and WM groups (*p* > 0.05) or CF and CM groups (*p* > 0.05) (Figure 2). 

**Figure 3:**
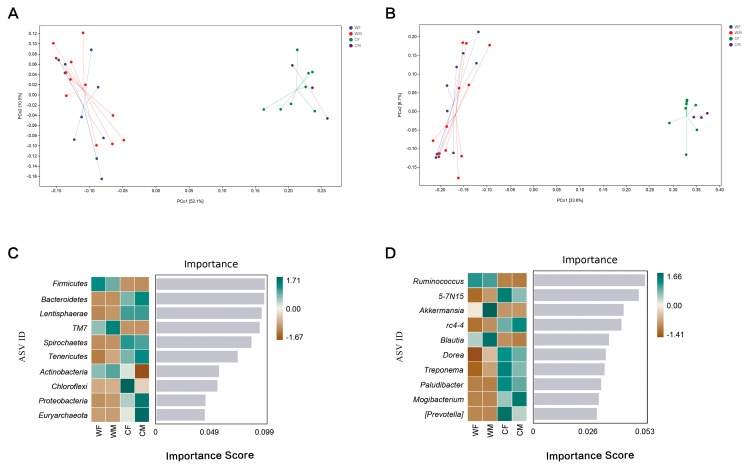
Faecal microbial structure of red deer. (**A**) Principal Coordinate Analysis based on weighted unifrac showing microbial samples clustered by sex and environment into different groups (in parenthesis, the explained variance of each axis; WF: wild female; WM: wild male; CF: captivity female; CM: captivity male). (**B**) Principal Coordinate Analysis based on unweighted unifrac showing microbial samples clustered by sex and environment into different groups (in parenthesis, the explained variance of each axis; WF: wild female; WM: wild male; CF: captivity female; CM: captivity male). (**C**) A random forest heatmap indicating intergroup differences at the phylum level. Importance means screening the most critical phylum or genus that leads to microbial structural differences between groups (WF, WM, CF, CM). (**D**) A random forest heatmap indicating intergroup differences at the genus level (WF: *n* = 10, WM: *n* = 12, CF: *n* = 8, CM: *n* = 3).

Wild and captive red deer also differed in beta-diversity. An PCoA plot based on the Unweighted Unifrac and Weighted Unifrac distance matrix revealed clear separation of the fecal microbiota between wild and captive red deer (Figure 3A). The results of a PCoA analysis showed that the fecal microbial structures of the CF and CM groups were more similar than those of the WF and WM communities (*F* = 13.82, *p* = 0.001; and unweighted: *F* = 5.983939, *p* = 0.001; Figure 3A; Supplementary Table S5).

A random forest analysis showed that Firmicutes and Bacteroidetes were the primary microorganisms that had differences between the wild and captive populations by (an importance > 0.1) (Figure 3C, D). This analysis indicated that there were significant differences in the abundances of Firmicute and Bacteroidetes between the four groups (an importance > 0.1), which were the primary phyla that caused differences in the microbial communities between groups (Figure 3C). *Ruminococcus*, *Treponema*, *Akkermansia*, a candidate genus *5-7N15* belonging to family Bacteroidaceae, and a candidate genus *rc4-4* belonging to family Peptococcaceae were the main genera that caused differences in microbial communities between sex and environment (importance > 0.04; Figure 3D).

### 3.5. Functional Modules of Fecal Microbial Communities 

**Figure 4:**
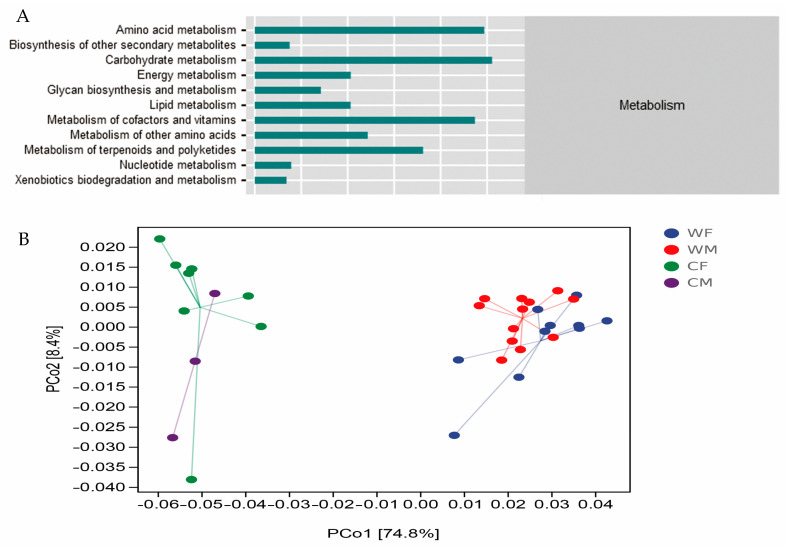
(**A**) Metabolic composition of secondary function pathways. (**B**) A PCoA plot based on weighted UniFrac distance metrics associated with a functional dataset. The colors represent sample sets of microbial functions (WF: *n* = 10, WM: *n* = 12, CF: *n* = 8, CM: *n* = 3).

Metabolism was found to be the most common function prediction performed on fecal microbial communities and included the most important pathways for microbial clustering (76.67%). The second pathway of metabolism included amino acid metabolism (17.26%), carbohydrate metabolism (17.85%), metabolism of cofactors and vitamins (16.57%), and metabolism of terpenoids and polyketides (12.66%) (Figure 4A). A PCoA analysis showed that the WF and WM groups had more similar microbial function clusters (Figure 4B).

**Figure 5:**
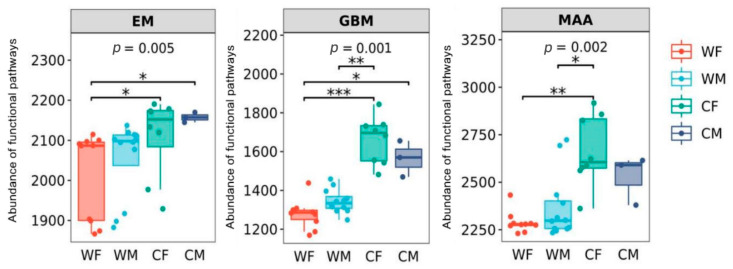
Microbial functions related to energy metabolism (EM), glycan biosynthesis and metabolism (GBM), and metabolism of other amino acids (MAA) grouped by sex and environment (WF: wild female; WM: wild male; CF: captivity female; CM: captivity male). * represents significant (*p* < 0.05), ** represents very significant (*p* < 0.01), and *** represents highly significant (*p* < 0.001) (WF: *n* = 10, WM: *n* = 12, CF: *n* = 8, CM: *n* = 3).

It was found that there were significant differences in the three metabolic pathways of glycan biosynthesis and metabolism (GBM), energy metabolism (EM), and metabolism of other amino acids (MAA) (*p* < 0.05) (Figure 5).

## 4. Discussion

This is the first study to apply high-throughput sequencing to describe the fecal bacterial microbiota of wild and captive red deer by sex. Analysis of the differences in fecal microbiota is a key step in releasing captive red deer to help expand the wild population. In general, the fecal bacterial microbiota of red deer was similar to that of other cervidae, such as elk (*Cervus canadensis*), white tailed deer (*Odocoileus virginianus)* [38], and white-lipped deer (*Cervus albirostris*) [39], at least at the bacterial phylum level, with high proportions of the phyla Firmicutes and Bacteroidetes. In the digestive tract of herbivores, the role of Firmicutes is mainly to decompose cellulose and convert it into volatile fatty acids, thereby promoting food digestion and host growth and development. The enrichment of Firmicutes plays an important role in promoting the ability of red deer to obtain abundant nutrients from food and, at the same time, affects the metabolic function of the fecal microbiota. Bacteroidetes can improve the metabolism of organisms, promote the development of the gastrointestinal immune system, participate in the body’s bile acid, protein, and fat metabolisms, and also have a certain regulatory effect on carbohydrate metabolism. It can also produce special glycans and polysaccharides, which have a strong inhibitory effect on inflammation [43]. Differences in microbiota may be explained by changes in diet. Data from previous local and overseas studies have shown that diet is the main factor affecting the gut microbiota in mammals [40]. It is likely that wild deer have a more varied diet, more than captive deer. These phyla, Firmicutes and Bacteroidetes, are involved in important processes such as food digestion, nutrient regulation and absorption, energy metabolism, and host intestinal defense against foreign pathogens [40,41,42].

Alpha diversity alterations may be attributed to differential diet or hormonal influences on the gut microbiota. Fecal microbiota richness in wild populations is higher than that in captive animals, such as the Tibetan wild ass (*Equus kiang*), bharal (*Pseudois nayaur*), Tibetan sheep (*Ovis arise*), and yak (*Bos mutus*) [44,45,46,47,48]. Nevertheless, other studies also found that captivity might increase the alpha diversity of fecal microbiota in most Cervidae compared with other animals, for example, sika deer (*Genus Cervus*), Père David’s (*Elaphurus davidianus*), and white-tailed deer (*Odocoileus virginianus*) [49,50]. It may be that some environmental stresses in the wild or the special structure of the stomach and intestines in these deer lead to decreased alpha diversity of fecal microbiota in wild deer [50]. This phenomenon needs further research to determine its cause. Our results showed that the richness of the fecal microbial community in wild red deer differed by sex (Figure 2). In wild deer, the microbiota diversity was higher for females than males. Microbial community alterations by sex could be attributed to hormonal [51]. The sampling time was during the gestation period of red deer. Levels of female growth hormone during pregnancy may affect the fecal microbiota. Reproductive hormones have also been associated with sex and gut microbial changes in wild animals [17,52,53]. Increased evidence indicates that sex steroid hormone levels are associated with the human gut microbiota [54,55]. Futher, Edwards et al. reported that estrogen and progesterone had an impact on gut function [56]. The captive deer also had the smallest sample size (*n* = 3 males and 8 females), which limited our ability to detect these differences.

In this study, the functional pathway composition of wild red deer is more similar (Figure 5B), which is completely opposite to the microbial structure (Figure 3A). The change in microbial structure does not necessarily lead to the change in function, which may be due to the same function in different microbial communities [57]. In recent years, studies have shown that gut microbiota are involved in various metabolic processes such as amino acids, carbohydrates, and energy, confirming their primary role in assisting host digestion and absorption [58]. It has also been found to be involved in environmental information processing, suggesting that the gut microbiota plays an important role in facilitating acclimation to changing environments [59]. The metabolism of gut microbiota is closely related to the feeding habits of the host. In the long-term evolution process, the gut microbiota will respond to changes in diet types or specific diets by adjusting the content of certain digestive enzymes [4,60]. Studies have shown that the decrease of fecal microbial diversity can lead to a reduction in the functional microbiota, in the efficiency of the microbiota, and in the resistance to pathogen invasion [61]. The decrease in fecal microbial diversity in captive populations resulted in a decrease in functional microbiota [61]. Ruminococcaceae and Lachnospiraceae are two of the most common bacterial families within the Firmicutes phylum [62]. It has been hypothesized that they have an important role as active plant degraders [63,64]. According to our results, the level of Ruminococcaceae in the captive groups is significantly lower than that in the wild group, which could suggest that the fiber-reduced diet in captivity is modifying the ability of the fecal microbiota to degrade recalcitrant substrates such as cellulose, hemicellulose, and lignocellulose, among others, that are commonly found on the main resources of the wild red deer diet. The captive deer’s consequent reduction of diet resources might trigger the decline of important metabolic pathways associated with nutrient use [64].

16S rRNA analysis constitutes a valuable and cost-efficient approach for surveillance and monitoring wild populations as well as captive individuals. Picrust2 prediction accuracy is dependent on the availability of closely related annotated bacterial genomes in the database and the phylogenetic distance from the reference genome. However, the prediction results are still uncertain, which does not mean that the correlation between the predicted genes and the real metagenome of the microbiota is 100% [65]. At present, due to the difficulty of cultivation, the mechanism by which some functional bacteria exert their effects remain unclear. Therefore, in the follow-up work, it is necessary to repeatedly cultivate the conditions of some intestinal anaerobic bacteria, the most extensive of which are Firmicutes and some Bacteroidetes. The microbiota was cultured in vitro by simulating the gut environment, and its functions were speculated and further verified in combination with multiple groups of studies (metagenomics, meta transcriptome, and proteome, etc.). At the same time, the unknown functional microbiota and its genome sequence information can be explored and studied. These works will help to understand the metabolic activities of the complex microbiota and further explore the host physiological processes involved in gut microbiota.

## 5. Conclusions

In conclusion, our study provided information on the structure and function of the fecal microbiome of red deer through the 16S rRNA gene of fecal samples. Comparing analyses identified significant variations of fecal microbiota composition and functions between captive and wild populations and also indicated that environment and sex have a great influence on these variations. These findings were of great significance for the reintroduction of captive red deer, given that the differences in fecal microbiota composition and functions between captive and wild red deer would greatly impact the ability of captive red deer to adapt to the wild environment. For further study, incorporating novel methods (e.g., transcriptome) to study the functional annotation of gene content and the functional traits of the host would be essential for better understanding the physiology and immunology of red deer.

## Figures and Tables

**Table 1:** Relative abundance > 1% at the genus level for four groups of red deer fecal microbiota.

Tax_Name	WF	WM	CF	CM
*Oscillospira*	4.71 ± 0.79	4.55 ± 0.73	4.55 ± 0.41	2.89 ± 0.31
*5-7N15*	3.81 ± 1.4	4.51 ± 1.53	4.51 ± 1.71	4.01 ± 1.29
*Ruminococcus*	3.05 ± 0.5	3.1 ± 0.56	3.1 ± 0.37	2.03 ± 0.6
*Roseburia*	2.27 ± 1.11	2.17 ± 1.08	2.17 ± 0.63	0.37 ± 0.35
*Clostridium*	1.55 ± 0.59	1.67 ± 1.01	1.67 ± 0.67	0.88 ± 0.68
*Prevotella*	1.32 ± 0.11	1.25 ± 0.12	1.25 ± 0.08	1.31 ± 0.31

## Data Availability

The datasets generated for this study can be found at NCBI/BioProject/PRJNA830551 (https://www.ncbi.nlm.nih.gov/bioproject/PRJNA830551 (accessed on 1 March 2023)).

## Supplementary Materials

The following supporting information can be downloaded at: https://www.mdpi.com/article/10.3390/ani13050929/s1, Figure S1: The rarefaction curve based on the Chao1 diversity index; Table S1: The amplification system of PCR based on 16S rRNA gene. Table S2: Individual information of 33 red deer. Table S3: The ratio and nutritional content of plants eaten by wild red deer of the Gaogestai Reserve in winter. Table S4: Nutritional content of the main food of captive red deer per 100 grams. Table S5: The results of PERMANOVA analysis based on weighted UniFrac and Unweighted UniFrac.
