# Supplementary material for: Alterations in Fecal Microbiota Linked to Environment and Sex in Red Deer (Cervus elaphus)
Source: Animals (Basel). 2023 Mar 4;13(5):929. doi: 10.3390/ani13050929 (PMC10000040; doi:10.3390/ani13050929)
Supplement: Supplementary file 1 [file animals-13-00929-s001.zip › Supplementary Table S1.pdf]

Table S1 The amplification system of PCR based on 16S rRNA gene.

Composition	Volum (μL)
Q5 high-fidelity DNA polymerase	0.25
5*Reaction Buffer	5
5* High GC Buffer	5
dNTP（10mM）	2
Template DNA	2
Forward primers（10uM）	1
Reverse primers（10uM）	1
water	8.75

Procedure：

98℃	5min	
98℃	30 s	25 cycles
53℃	30 s	25 cycles
72℃	45 s	25 cycles
72℃	5 min	
12℃	∞	
