# Supplementary material for: Alterations in Fecal Microbiota Linked to Environment and Sex in Red Deer (Cervus elaphus)
Source: Animals (Basel). 2023 Mar 4;13(5):929. doi: 10.3390/ani13050929 (PMC10000040; doi:10.3390/ani13050929)
Supplement: Supplementary file 1 [file animals-13-00929-s001.zip › Supplementary Table S2.pdf]

Table S2 Individual information of 33 red deer

Number	Sample Number	Sex	location	Diet	Study sites	sampling time
1	WF1	female	Gaogestai National Nature Reserve in Chifeng, Inner Mongolia,	*Ulmus pumila*、*Salix spp.*、 *Tripolium vulgare*、 *Armeniaca sibirica*、 *Betula platyphylla*、*Spiraea salicifolia*、 *Larix gmelinii*、 *Lonicera chrysantha*、 *Rosa davurica*、 *Polygonum divaricatum*、 *Ostryopsis davidiana*、 *Rhododendron dauricum*、 *Lespedeza bicolor*、 *Caragana microphylla*	The Gaogestai Reserve in Chifeng, Inner Mongolia.	2019.3.9
2	WF2	female				2019.3.8
3	WF3	female				2019.3.13
4	WF4	female				2019.3.11
5	WF5	female				2019.3.10
6	WF6	female				2018.12.17
7	WF7	female				2018.12.22
8	WF8	female				2018.12.22
9	WF9	female				2018.12.19
10	WF10	female				2018.12.19
11	WM1	male				2019.3.8
12	WM2	male				2019.3.11
13	WM3	male				2019.3.9
14	WM4	male				2019.3.13
15	WM5	male				2019.3.10
16	WM6	male				2019.3.13
17	WM7	male				2018.12.22
18	WM8	male				2018.12.22
19	WM9	male				2018.12.18
20	WM10	male				2018.12.19
21	WM11	male				2018.12.18
22	WM12	male				2018.12.20
23	CF1	female	Hanshan Forest Farm in Chifeng, Inner Mongolia,	Corn、Soya bean、wheat bran、Elm leaves、Mongolian oak leaves	The Han Mountain Farm in Chifeng City, Inner Mongolia (adjacent to Gaogestai Nature Reserve).	2019.12.10
24	CF2	female				
25	CF3	female				
26	CF4	female				
27	CF5	female				
28	CF6	female				
29	CF7	female				
30	CF8	female				
31	CM1	male				
32	CM2	male				
33	CM3	male				
