# Supplementary material for: Alterations in Fecal Microbiota Linked to Environment and Sex in Red Deer (Cervus elaphus)
Source: Animals (Basel). 2023 Mar 4;13(5):929. doi: 10.3390/ani13050929 (PMC10000040; doi:10.3390/ani13050929)
Supplement: Supplementary file 1 [file animals-13-00929-s001.zip › Supplementary Table S3.pdf]

Table S3 The ratio and nutritional content of plants eaten by wild red deer of the Gaogestai Reserve in winter.

Plant species	Crude protein(%)	Energy content(J/g)	Fiber (%)
Ulmus pumila	3.4	15498.06	66.54
Salix spp.	5.41	20038.91	60.49
Tripolium vulgare	4.27	18720.83	63.95
Armeniaca sibirica	3.76	16721.71	57.05
Betula platyphylla	5.28	21833.83	62.67
Spiraea salicifolia	4.18	22698.18	58.85
Larix gmelinii	1.26	16629.28	50.32
Lonicera chrysantha	5.37	18810.99	51.44
Rosa davurica	3.59	16201.82	58.54
Polygonum divaricatum	3.51	19038.82	66.34
Ostryopsis davidiana	3.54	17630.33	44.54
Rhododendron dauricum	3.11	17343.23	62.21
Lespedeza bicolor	5.29	12029.88	68.86
Caragana microphylla	4.25	17320.63	58.42
