# Supplementary material for: Alterations in Fecal Microbiota Linked to Environment and Sex in Red Deer (Cervus elaphus)
Source: Animals (Basel). 2023 Mar 4;13(5):929. doi: 10.3390/ani13050929 (PMC10000040; doi:10.3390/ani13050929)
Supplement: Supplementary file 1 [file animals-13-00929-s001.zip › Supplementary Table S4.pdf]

Table S4 Nutritional content of the main food of captive red deer per 100 grams.

Species	Protein(%)	fiber(%)	Carbohydrate(%)	Fat(%)
Corn	7	4.4	66	2.7
Soya bean	31	13.1	15	10.3
wheat bran	13	20	55	3
Elm leaves	5.2	7.5	37	4
Mongolian oak leaves	11.2	8.3	40	5.6
