# Supplementary material for: Alterations in Fecal Microbiota Linked to Environment and Sex in Red Deer (Cervus elaphus)
Source: Animals (Basel). 2023 Mar 4;13(5):929. doi: 10.3390/ani13050929 (PMC10000040; doi:10.3390/ani13050929)
Supplement: Supplementary file 1 [file animals-13-00929-s001.zip › Supplementary Table S5.pdf]

Table S5 The results of PERMANOVA analysis based on weighted UniFrac and Unweighted UniFrac

weighted UniFrac

Group1	Group2	Sample#size	Permutations	pseudo#F	p#value	q#value
all	-	33	999	13.818965	0.001	-
WF	WM	22	999	0.79107	0.547	0.547
WF	CF	18	999	21.734279	0.001	0.003
WF	CM	13	999	14.602901	0.005	0.0075
WM	CF	20	999	24.696271	0.001	0.003
WM	CM	15	999	16.786507	0.003	0.006
CF	CM	11	999	1.259629	0.203	0.2436

Unweighted UniFrac

Group1	Group2	Sample#size	Permutations	pseudo#F	p#value	q#value
all	-	33	999	5.983939	0.001	-
WF	WM	22	999	0.880224	0.756	0.756
WF	CF	18	999	9.363555	0.001	0.003
WF	CM	13	999	7.416048	0.004	0.006
WM	CF	20	999	9.362129	0.001	0.003
WM	CM	15	999	6.704129	0.003	0.006
CF	CM	11	999	1.38619	0.073	0.0876
