# Supplementary figures and images for: Alterations in Fecal Microbiota Linked to Environment and Sex in Red Deer (Cervus elaphus)
Source: Animals (Basel). 2023 Mar 4;13(5):929. doi: 10.3390/ani13050929 (PMC10000040; doi:10.3390/ani13050929)

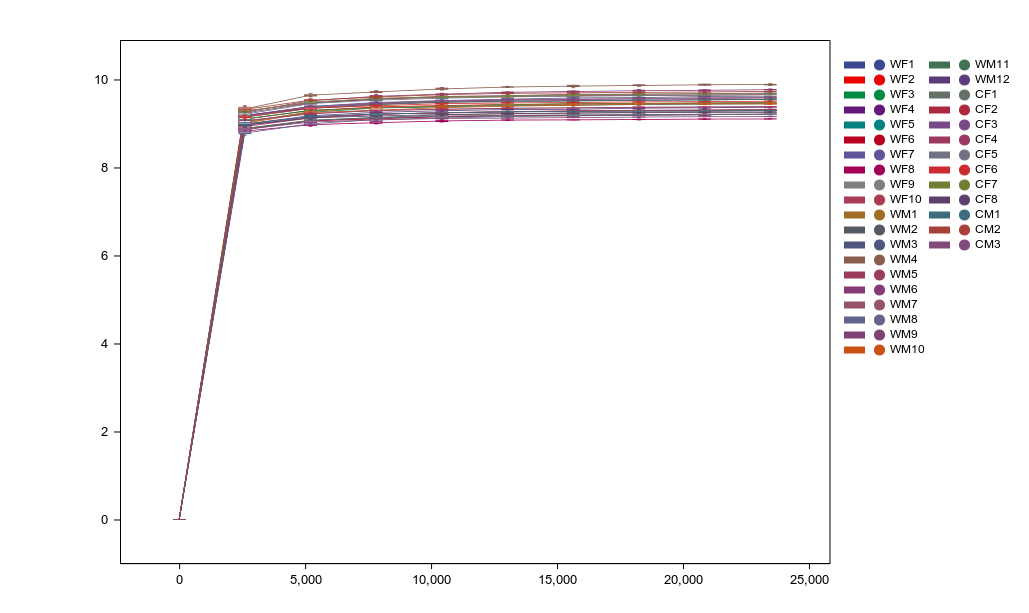

Supplement: Supplementary file 1 [file animals-13-00929-s001.zip › Supplementary Figure S1.png]
